# Supplementary material for: Physalis angulata Calyces Modulate Macrophage Polarization and Alleviate Chemically Induced Intestinal Inflammation in Mice
Source: Biomedicines. 2020 Feb 5;8(2):24. doi: 10.3390/biomedicines8020024 (PMC7168001; doi:10.3390/biomedicines8020024)
Supplement: Supplementary file 1 [file biomedicines-08-00024-s001.pdf]

Physalis angulata calyces modulate macrophage polarization and alleviate chemically induced intestinal inflammation.

David Rivera, Yanet Ocampo and Luis A. Franco

1. Materials and Methods

1.1. Preparation of the dichloromethane fraction from P. angulata

The calyces from *P. angulata* were cleaned and dried at room temperature for 2–3 days. The dried material was powdered using a lab blender (Waring®, New Hartford, CT, USA) to obtain a fine powder (233.7 g) that was extracted with 3 L of ethanol (Sigma-Aldrich, St Louis, MO, USA) by maceration at room temperature for 24–48 h (3 times). The ethanolic extract was filtered (Whatman filter paper, pore size: 11 µM, GE Healthcare, China) and the solvent evaporated to dryness under reduced pressure at 37 ± 2 °C using a rotary evaporator (Heidolph VV 2000, Heidolph, Kelheim, Germany) to obtain 31 g of total extract (13.26% yield). This extract (20 g) was further fractioned using liquid-liquid partition with petroleum ether, dichloromethane, and methanol:water (9:1) (Merck, Darmstadt, Germany). The dichloromethane fraction (PADF) was dried in a rotary evaporator and weighted to calculate a yield of 31.49%.

For cell culture experiments, PADF was dissolved in DMSO and diluted in DMEM so that the final concentration of vehicle was lower than 0.1% (*v*/*v*). For animal experiments, PADF was co-precipitated with polyvinylpyrrolidone (PVP K-30, Sigma-Aldrich, St Louis, MO, USA) in a 1:4 (*w*/*w*) ratio using the solvent evaporation technique.

1.2. HPLC Fingerprint Analysis

PADF was dissolved in HPLC-graded methanol (20 mg/mL), treated for 1–2 min in an ultrasonic bath, and filtered through 0.45 µm syringe filters (Acrodisc® GHP, PALL Life Science, Ann Arbor, MI, USA). The HPLC fingerprint analysis was performed with an HPLC instrument (Hitachi Elite LaChrom, Japan) using a Mediterranea SEA C_{18} column (25 × 0.46 cm, 5 µm, Teknocroma, Spain), as well as water (Solvent A) and acetonitrile (Solvent B). The solvent program was set as follows: 7–50% B (0–5 min), 50–70% B (5–25 min), 70% B (25–30 min), 70–90% B (30–40 min), 90–100% B (40–55 min), 100% B (55–65 min), 100–7% B (65–70 min). The injection volume was 10 µL, the flow rate was 1 mL/min, the column temperature was maintained at 40 °C, and the detection was performed at 222 nm.

1.3. Quantification of total sucrose esters content

The total content of sucrose esters in PADF was determined spectrophotometrically using the procedure described by [1]. The sample (1.2 mg) was dissolved in ethanol (0.1 mL) to obtain a stock solution that was further diluted in 0.9 mL of water (1.2 mg/mL). Then 2.5 mL of anthrone reagent (0.2% in concentrated sulfuric acid) were added slowly to 0.5 mL of the stock solution, while avoiding overheating using an ice bath. Subsequently, the reaction was kept at 70 °C for 30 min and the optical density (OD) was measured at 625 nm using a microplate reader (Multiscan EX Thermo®). The total sucrose esters content (%) was calculated using a sucrose standard curve (0.019–1.20 mg/mL) and compared to a reference of a mixture of natural sucrose esters (peruvioses A and B) isolated from *Physalis peruviana* calyces.

1.4. Determination of total withanolides content

A modified spectrometric method was used to determine total withanolides content in PADF as described by [2]. For this purpose, 0.1 mL of PADF (10 mg/mL in methanol) were mixed with 0.2 mL

of glacial acetic acid and 2.1 mL of color reagent (1.72% ferric chloride hexahydrate and 8% orthophosphoric acid in concentrated sulfuric acid) avoiding overheating with an ice bath. After 5 min, the OD_{540} of the samples was measured using a microplate reader and the concentration of withanolides was calculated using cholesterol as standard (0–2000 mg/mL).

1.5. Production of NO, Urea and IL-10 by activated macrophages

RAW 264.7 macrophages (750,000 cells/well) were seeded in 6-well plates for 48 h, then treated with PADF (0–12.5 μg/mL) for 1 h, and stimulated with LPS (1 μg/mL) or IL-4 (40 ng/mL) to induce an inflammatory M(LPS) or an alternative M(IL4) profile, respectively. Twenty four hours after stimulation, the supernatants were collected and stored at -80 ºC. The release of nitric oxide was determined spectrophotometrically by the accumulation of nitrite (NO_2^-), using the Griess reaction [3]. Briefly, 100 μL of cell culture medium was mixed with 100 μL of Griess reagent (1:1 mixture of 0.1% N-(1-naphthyl) ethylenediamine dihydrochloride and 1% sulfanilamide in 5% H_3PO_4), and incubated at room temperature for 5 min. The OD_{550} of the samples was measured using a microplate reader and compared with a standard curve prepared with $NaNO_2$ (1–200 μM). The levels of urea were determined using the UREA-LQ® enzymatic kit (SPINREACT, Girona, Spain) according to the manufacturer's instructions. Final results were expressed as mg/dL. Quantification of interleukin (IL)-10 was performed using a commercial ELISA kit (eBioscience, San Diego, CA, USA) as instructed by the manufacturer. Final results were expressed as pg/mL.

2. Results

2.1. HPLC fingerprint of P. angulata dichloromethane fraction (PADF)

The HPLC fingerprint analysis revealed the presence of 14 characteristic peaks, with proper intensity and good resolution, representing 73% of the total area of the chromatogram (**Figure S1**).

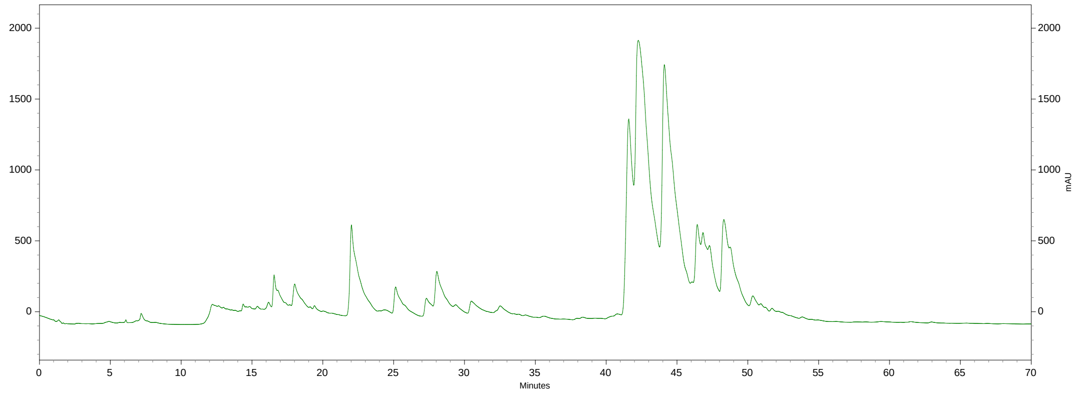

Figure S1. HPLC fingerprint chromatogram of *P. angulata* fraction (PADF). The fingerprint was acquired using an HPLC instrument (Hitachi Elite LaChrom) equipped with a diode array detector (DAD) and the detection recorded at 222 nm.

Peaks 3, 6, 9, 10, 11, and 14 represent the 58.9% of the total area; therefore they were classified as the major metabolites in PADF (Table S1). Peak 11 exhibited one of the most important signals, corresponding to 18.15% of the total area, and presenting a reproducible retention time, stable area and proper symmetry, hence it was selected as reference peak. The chemical identity of these characteristics peaks is unknown since no standard compounds are available to study the *Physalis* genus. However, the method developed for the HPLC fingerprint analysis is useful for quality control purposes, for instance to guarantee proper fractionation, to standardize new extraction methods, and to identify the chemical changes that promote a shift in bioactivity.

Table S1. Retention time of the main peaks of the HPLC fingerprint of *P. angulata* fraction (PADF).

Peak	Retention Time (min)	Area
**1**	16.81	1.31%
**2**	18.01	2.12%
**3**	22.02	5.52%
**4**	24.35	0.68%
**5**	25.14	2.40%
**6**	28.05	2.82%
**7**	29.38	1.03%
**8**	30.48	1.86%
**9**	41.59	6.78%
**10**	42.27	21.08%
**11**	44.11	18.15%
**12**	46.83	2.63%
**13**	47.29	2.99%
**14**	48.76	3.84%
**Total**	-	72.53%

In fact, this method was useful to compare the fingerprint from fractions prepared from plants collected in different seasons (Dry season 2016 Vs. Rainy season 2013, Figure S2) to identify that the substantial reduction in the area of peaks 10–14 does not affect the bioactivity of PADF against nitric oxide production by LPS-stimulated RAW264.7 (data not shown).

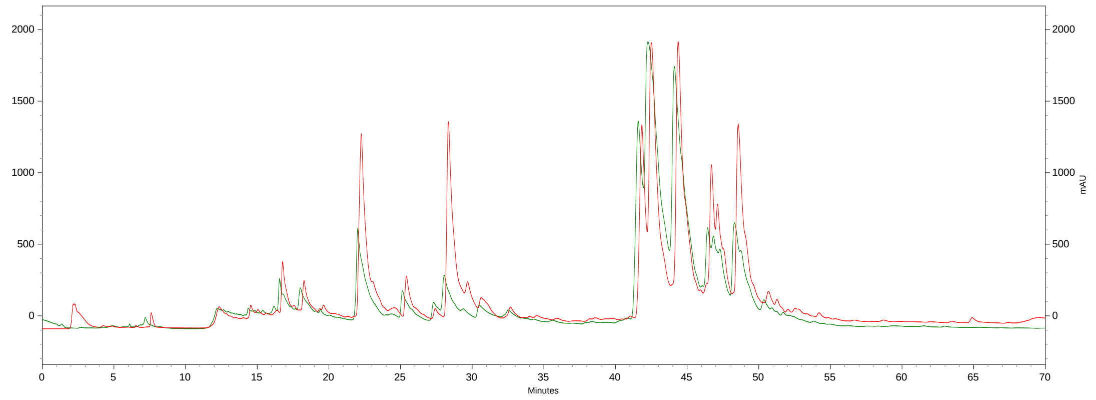

Figure S2. Comparison of the HPLC fingerprints of *P. angulata* fractions (PADF). Fractions were obtained during dry season (green) or rainy season (red) and their fingerprints were acquired using the HPLC method described before.

2.2. Total content of sucrose esters and withanolides

The results of secondary metabolites quantification are summarized in Table S2. It can be noticed that the content of glycosides is significantly high in the test fraction, whereas the content of withanolides was surprisingly low. Most likely these glycosides originate from sucrose esters since phenolic compounds were detected in low abundance (Total Phenolics Content= 8.78 ± 0.17 mg/g – Folin-Ciocalteu) and flavonoids were not detectable (Aluminum trichloride method). Therefore, calyces of *P. angulata* might be considered an important source of sucrose esters.

Table S2. Metabolite content of *Physalis angulata* dichloromethane fraction (PADF).

Sample	Total Sucrose Content	Total Withanolides Content
PADF	337.2 ±9.52 mg/g	5.11 ±1.63 mg/g
Peruvioses A and B	450.3 ±9.48 mg/g<br>(Expected: 481.07 mg/g)	---

2.2. Effect of P. angulata fraction (PADF) in the release of pro-inflammatory and anti-inflammatory markers by activated macrophages

Supernatants from inflammatory M(LPS) or alternative M(IL4) macrophages were studied to confirm the effect of PADF in their polarization. Results corroborated that the test fraction inhibited the production of nitrite by M(LPS), but did not increase the levels of urea in the case of M(IL-4) (**Figure S3**). On the other hand, when studying IL-10, it was noticed that the test fraction significantly inhibited its production by M(LPS) but had no effect in M(IL-4).

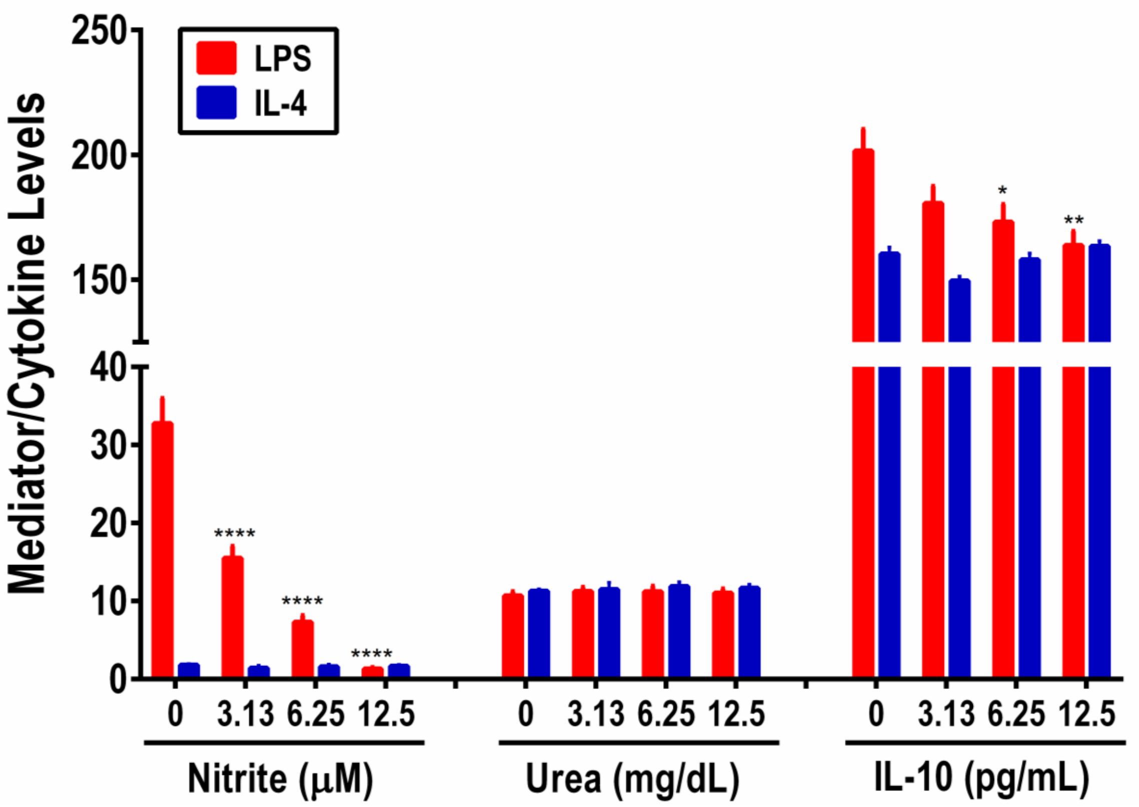

Figure S3. *P. angulata* fraction (PADF) regulates inflammatory macrophages [M(LPS)] but does not affect significantly the polarization induced by IL-4 [M(IL-4)]. Levels of nitrite, urea, and IL-10 were determined using the Griess reaction, indirect enzymatic detection or ELISA, as fully described in the supplementary methods. Results are expressed as the mean ± SEM from three independent experiments (n = 6–10). *p < 0.05, **p < 0.01, ****p < 0.0001 (in comparison to LPS group) and +p < 0.05, + + p<0.01 (in comparison to IL-4 group), as calculated by ANOVA and Dunnet's multiple comparisons test.

References

1. Mima, H.; N. Kitamori. Chromatographic analysis of sucrose esters of long chain fatty acids. *J. Am. Oil Chem. Soc.* **1964**, *41*, 198–200.
2. Devkar, S.T., et al., Effect of macronutrient deficiency on withanolides content in the roots of Withania somnifera and its correlationship with molybdenum content. *Pharm. Biol.* **2015**, *53*, 518–523.
3. Green, L.C., et al., Analysis of nitrate, nitrite, and nitrate in biological fluids. *Anal. Biochem.* **1982**, *126*, 131–138.
